# Diabetes Mellitus, Edentulism, and Trajectory of Cognitive Decline Among Older Adults

**DOI:** 10.1093/geroni/igab046.791

**Published:** 2021-12-17

**Authors:** Bei Wu, Chenxin Tan, Brenda Plassman, Frank Sloan, Mark Schwartz, Samrachana Adhikari, Angela Kamer, Huabin Luo

**Affiliations:** 1 New York University, New York, New York, United States; 2 Duke University, Durham, North Carolina, United States; 3 New York University, NYC, New York, United States; 4 East Carolina University, Greenville, North Carolina, United States

## Abstract

We examined the impact of diabetes mellitus (DM) and edentulism on the trajectory of cognitive decline, using the Health and Retirement Study. We analyzed self-reported DM and edentulism collected in 2006 and cognition data from 2006 and its follow up waves through 2018. Among 15,709 eligible participants age 50+ in 2006, 65.96% had neither DM nor edentulism (Group 1), 15.12% had DM alone (Group 2), 13.79% had edentulism alone (Group 3), and 5.12% had both conditions (Group 4). Results from linear mixed-effects models show that in comparison to Group 1, individuals in Group 4 had the lowest level of cognitive function, followed by those in Group 3 and Group 2. Group 4 had a modestly faster rate of cognitive decline (p=0.052). This study illustrates that co-occurrence of DM and edentulism has a higher risk of more rapid cognitive decline with advancing age than the presence of each condition alone.

